# Efficient *In Silico* Saturation Mutagenesis of a Member of the Caspase Protease Family

**DOI:** 10.1021/acs.jcim.0c01216

**Published:** 2021-02-11

**Authors:** Christoph Öhlknecht, Sonja Katz, Christina Kröß, Bernhard Sprenger, Petra Engele, Rainer Schneider, Chris Oostenbrink

**Affiliations:** † Institute of Molecular Modeling and Simulation, University of Natural Resources and Life Sciences, Vienna A-1190, Austria; ‡ Austrian Centre of Industrial Biotechnology, Petersgasse 14, Graz 8041, Austria; § Institute of Biochemistry and Center of Molecular Biosciences Innsbruck, University of Innsbruck, Innsbruck 6020, Austria

## Abstract

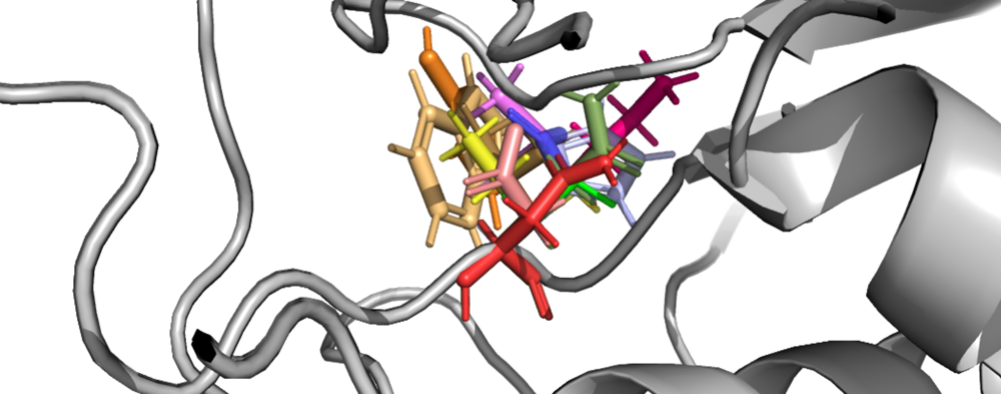

Rational-design methods have proven to be a valuable toolkit in the field of protein design. Numerical approaches such as free-energy calculations or QM/MM methods are fit to widen the understanding of a protein-sequence space but require large amounts of computational time and power. Here, we apply an efficient method for free-energy calculations that combines the one-step perturbation (OSP) with the third-power-fitting (TPF) approach. It is fit to calculate full free energies of binding from three different end states only. The nonpolar contribution to the free energies are calculated for a set of chosen amino acids from a single simulation of a judiciously chosen reference state. The electrostatic contributions, on the other hand, are predicted from simulations of the neutral and charged end states of the individual amino acids. We used this method to perform *in silico* saturation mutagenesis of two sites in human Caspase-2. We calculated relative binding free energies toward two different substrates that differ in their P1′ site and in their affinity toward the unmutated protease. Although being approximate, our approach showed very good agreement upon validation against experimental data. 76% of the predicted relative free energies of amino acid mutations was found to be true positives or true negatives. We observed that this method is fit to discriminate amino acid mutations because the rate of false negatives is very low (<1.5%). The approach works better for a substrate with medium/low affinity with a Matthews correlation coefficient (MCC) of 0.63, whereas for a substrate with very low affinity, the MCC was 0.38. In all cases, the combined TPF + OSP approach outperformed the linear interaction energy method.

## Introduction

Traditional directed-evolution methods utilize a two-step protocol with an initial generation of a rich library by random mutagenesis^1^ and then identifying those library members that show improvements in the desired functions.^2^ Such a randomly generated library must be huge in order to be relevant, and both generating and screening such a library is expensive. Novel methods have advanced in recent years to substitute the random mutagenesis by knowledge-based design.^3^ These modern design methods are fit to allow smaller libraries but preserve or even enhance their relevance. They do so by replacing the random components by information about the structure and function of protein sequences, usually supported by computational algorithms such as QM or MD calculations or machine-learning methods.^4−6^ Rational methods can improve the productivity toward the engineered protein in two, usually in sequential steps: (1) locating potential target sites for mutation and (2) narrowing the list of possible amino acids for substitution.

We recently provided a successful example of such a rational design procedure.^7^ In this work, statistical and computational methods were jointly applied to engineer human Caspase-2 (Casp-2). The task was to create a biochemical scissor that is fit to cleave fusion tags from a wide variety of proteins. In close proximity to the active site, two point mutations were located to yield a more promiscuous S1′ subsite. After locating potential target sites through a combination of statistical methods with structural information, changes in binding free energies upon mutation were assessed using MD simulations. In short, free energies were calculated with the thermodynamic integration (TI) approach^8^ along progressive perturbations using a λ-dependent Hamiltonian of the system. The information-based hypotheses were confirmed experimentally through measurements of cleavage times and Michaelis Menten parameters. These alchemical methods–if done correctly–have been proven to be very accurate and reliable.^9−13^ The drawback of these calculations is their high cost in terms of computational power.

Information-based mutagenesis, although more efficient than random mutagenesis, still leaves a huge part of the protein sequence space unknown. On the experimental side, efficient protocols have been described to introduce mutations with all possible amino acids, also known as saturation mutagenesis.^14−16^ A novel protocol for *in silico* saturation mutagenesis was described recently.^17^ Here, the non-bonded contributions to the binding free energy are split up into two independent simulation steps for the non-polar (Lennard–Jones, LJ) contributions and the polar (Coulomb) contributions. For the non-polar contributions, a reference state is designed such that it samples relevant conformations to multiple end states. This reference state does not represent a physical molecule and the amount of relevant configurations sampled can be enhanced through a soft-core potential energy function.^18,19^ From this state, the free-energy differences to multiple end states can be calculated in a single step using the one-step perturbation (OSP) approach.^20−22^ The polar contribution to the free energies are calculated in a two-step charging process where only both end states have to be described explicitly. To approximate a full thermodynamic–integration profile, a cumulant expansion to determine the second derivatives of the free energy is utilized in the third-power fitting (TPF) method.^23^

**Figure 1:**
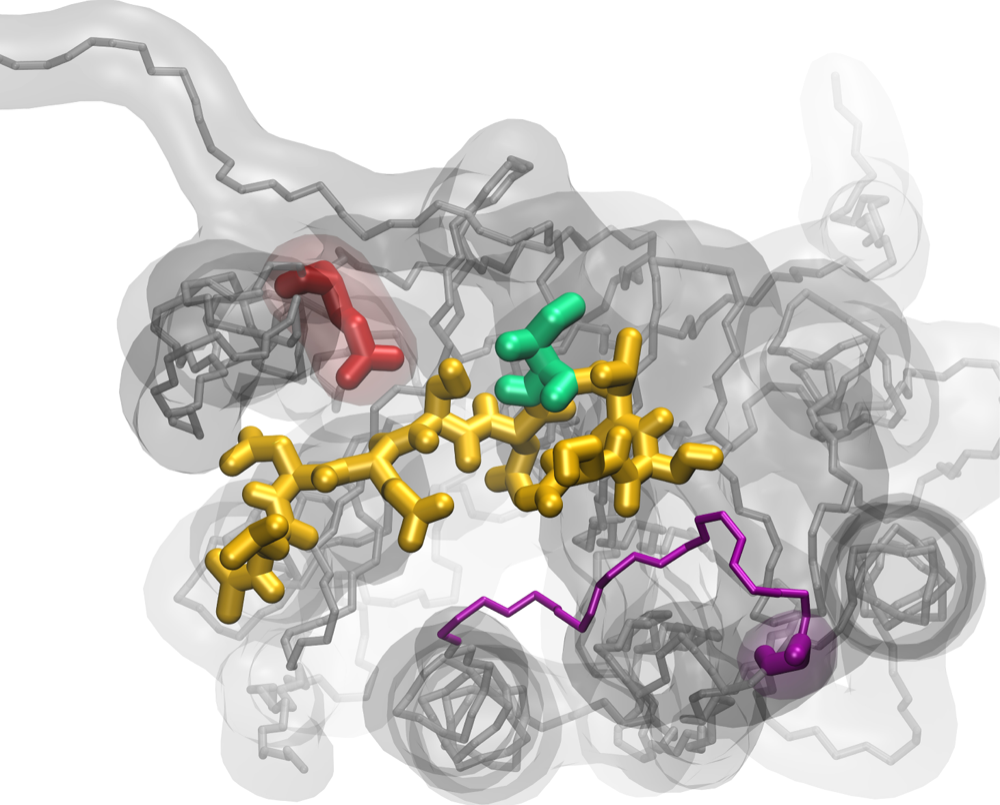
Snapshot of the Casp-2 active site with visualization of the entire substrate (orange), the P1′ site (green, here: Ile) and the two sites of point mutations E105 (red) and G171 (purple). While E105 is part of the substrate-binding cavity, the G171 site is not directly interacting but part of a binding loop that spans the entire binding cavity (purple stick representation of the backbone atoms).

Within this study, this *in silico* saturation mutagenesis method was used to explore the protein sequence space for two point mutations of E105 and G171. Both amino acid positions are in close proximity to the active site of Casp-2 (Figure 1) and were found to impact the specificity of the S1′ pocket.^24^ To develop a versatile protease that can cleave fusion tags from the N-terminus of any protein, the general aim was to identify mutations that enhance the promiscuity of the S1′ binding pocket.^7^ To achieve this, we calculated changes in the binding free energy of a model substrate upon mutation of both sites while keeping the respective other site unmutated. All calculations were performed twice using two different P1′ amino acids in the substrate, Ile and Pro. These two amino acids were chosen because branched, apolar amino acids and Pro are known to be weak binders of the S1′ site as can be found in the Merops Database.^7,25^ All predictions that are based on these free energies were validated by comparing to data from random saturation mutagenesis experiments. Furthermore, the predictions from this combined OSP + TPF approach were compared to predictions obtained from the linear interaction energy (LIE) approach.

## Methods

### MD Simulations

#### General Information

All simulations were run using the GROMOS11 molecular simulation software (http://www.gromos.net).^26^ Molecular interactions were described through the GROMOS 54A8 force field.^27^ For all simulations, water was treated explicitly and implemented by means of the three-site simple point charge model.^28^ Simulations were carried out under periodic boundary conditions (PBCs) based on rectangular computational boxes with at least 0.8 nm between any protein atom and the nearest box wall. The equations of motion were integrated using the leap-frog scheme.^29^ Bond vibrations were constrained using the SHAKE algorithm^30^ with a relative geometric tolerance of 10^–4^. The center of mass translation of the computational box was removed every 2 ps. The temperature and pressure were maintained at 298.15 K and 1 atm by weak coupling^31^ using a coupling time of τ_*T*_ = 0.1 ps and τ_*P*_ = 0.5 ps and an isothermal compressibility of 7.624 × 10^–4^ (kJ mol^–1^ nm^–3^)^−1^.^31^ Electrostatic interactions were calculated using a Barker–Watts reaction field (BM) scheme^32,33^ with a value of ϵ_BW_ = 61.^34^ Nonbonded interactions were calculated using a molecular twin-range charge-group cut-off scheme. The cutoff used for the short-range pairlist construction was set to 0.8 nm and the cutoff used for the long-range interactions was set to 1.4 nm. Interactions within the short range were calculated at every time step from a pairlist that was updated every 10 fs. At pairlist updates, interactions up to the long-range cutoff were computed and kept constant, as appropriate for simulations with the GROMOS force field.^35,36^

#### Preparation of the Protein

The Caspase-2 crystal structure in complex with the inhibitor *N*-acetyl-l-leucyl-l-α-aspartyl-l-α-glutamyl-l-seryl-l-aspartic aldehyde (PDB ID:1PYO)^37^ was retrieved from the PDB data bank (http://www.rcsb.org).^38^ Caspase-2 was resolved as a functional dimer, with a disulfide bridge linking the two monomers. The inhibitor was extended after the C-terminus by a chain of the sequence Ile-Val-Ser-Ser and Pro-Val-Ser-Ser to span the entire active site using the MOE 2017 loop modeler.^39^ From this loop modeler, the three structures with the highest scores were chosen as representative substrate starting structures. Subsequently, 120 ns long molecular dynamics (MD) simulations (40 ns per substrate starting structure) were performed. The last 30 ns of the individual simulations was used to find a representative substrate starting structure for the subsequent free-energy calculations. This was done by clustering using the algorithm by Daura *et al.*(40) with a cut-off distance of 0.23 nm. As a starting structure for all following simulations, one representative structure was chosen from the dominant cluster.

**Figure 2:**
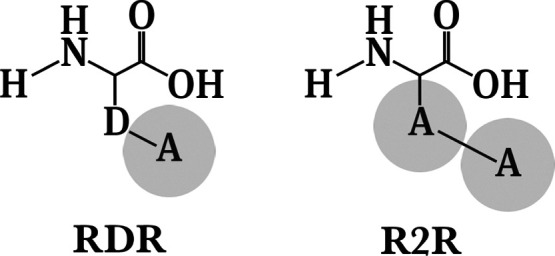
Structures of the two reference states used for the OSPs. Atom D is a non-interacting dummy atom while atom A represents a soft-core particle.

Regarding simulations of the reference states, all simulations were executed twice with two different reference states, one with two soft spheres (R2R) and one with a non-interacting dummy atom and one soft sphere (RDR) (Figure 2). These reference states (denoted R4 and R5 in Jandova *et al.*) were chosen as they showed the closest match to the more elaborate TI method, relative to alternative reference state molecules.^17^ The LJ parameters for the soft reference atoms were set to (*C*6)^1/2^ = 0.27322 (kJ mol^–1^ nm^6^)^1/2^ and (*C*12)^1/2^ = 0.056143 (kJ mol^–1^ nm^12^)^1/2^. This yields an effective VdW radius of 0.662 nm for the reference atoms. An LJ soft-core parameter of 1.51 was set for the reference atoms.^18,19^ The Cα–D and D–A bond lengths in RDR were set to 0.153 nm, while in R2R, the Cα–A bond length was set to 0.252 nm and the A–A bond length was set to 0.351 nm. The reference states were modeled into the protein at positions E105 and G171 such that the atoms of the backbone overlapped. This was done for both dimers. After energy minimization using the steepest descent algorithm, the entire system was equilibrated. The velocities were randomly assigned at 60 K and solute atoms were positionally restrained using a force constant of 2.5 × 10^4^ kJ mol^–1^. The system was heated up to 300 K in five discrete steps for 0.4 ns each, resulting in a total equilibration time of 2 ns. In each step, the force constant was lowered by 1 order of magnitude. The subsequent production runs were performed for 50 ns.

#### Free-Energy Calculations

**Figure 3:**
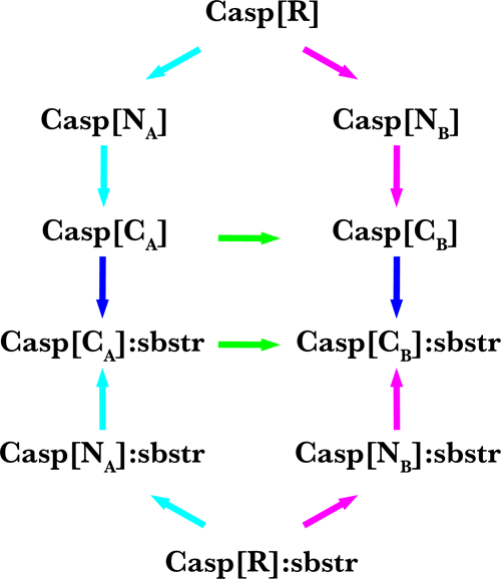
Thermodynamic cycle to calculate meaningful relative binding free energies from OSP of a reference state (R) and third-power fitting between neutral (N) and charged (C) states. Free energies along the black arrows were calculated from the simulations (OSP: R → N; TPF: N → C). Free energies along the blue arrows are binding free energies between the Caspase-2 (Casp) and its substrate (sbstr). The free-energy differences along the green arrows can be calculated from the differences between the cyan and magenta arrows. Relative free energies between the mutants (A,B) of the protein can be calculated from the difference between the two green arrows. The free-energy difference between the two green arrows (non-physical path) is the same as between the two blue arrows (physical path).

Relative binding free energies for all point mutations Glu105Xxx and Gly171Xxx were calculated from a set of OSP + TPF calculations using a thermodynamic cycle (Figure 3). Free energies of charging for amino acids bearing a net charge (as calculated from the TPF simulations) are artifacted raw free energies and need to be corrected using a set of correction terms. All employed methods are described in the following paragraphs.

#### One-step Perturbation

In order to predict the free-energy changes between the Hamiltonian of the reference states and the Hamiltonian of the end states (where “end state” refers to a physical amino acid), meaningful conformations of amino-acid side chains were fitted onto the trajectory with the reference states. These conformations of side chains were taken from a conformational library described in Jandova *et al.*(17) and used for both reference states, R2R and RDR. In the fitting procedure, the C_α_ atoms of the reference state and the amino acid conformation were aligned and then the side chain of the amino acid conformation was rotated such that the distances between the center of geometry of the side chain and the soft atoms were minimized. This procedure was repeated for each of the 25,000 conformations from the reference state simulation and for five different conformations per amino acid, as taken from the conformational library. The free-energy changes between the reference state R and the individual neutral, physical states N_*i*_ were calculated using the Zwanzig equation
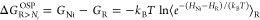
1where *H*_Ni_ refers to the Hamiltonian of the neutral, physical state N_*i*_, *H*_R_ refers to the Hamiltonian of the reference state, and ⟨...⟩_R_ denotes an ensemble average from a simulation of the reference state. *k*_B_*T* is the Boltzmann constant multiplied by the absolute temperature. To take into account the different relative occurrences P_*i*_ of the individual conformations, these were added to the free energies from OSP as
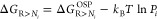
2

Finally, the resulting free energies from all *n* = 5 individual physical states were exponentially averaged
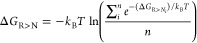
3

The procedure for Gly was different since this amino acid lacks a side chain. Here, free energies for removal of the side chains of the reference states were calculated using the OSP method to yield appropriate estimates. All free energies were calculated for both dimers of the protease and averaged. For comparisons against experimental data, the values of the two different tautomers of Histidine were also averaged.

#### Third-Power Fitting

The free-energy change between two end states, for example, with neutral and charged side chains, can be calculated formally exactly *via* the TI approach^8^
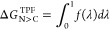
4with
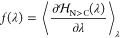
5with λ ∈ [0, 1] being the coupling parameter and ⟨...⟩_λ_ denotes ensemble averaging from a simulation at a discrete λ. The Hamiltonian of the system can be defined for any intermediate state between the neutral and charged end states (*vs*) using λ
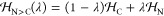
6

This property can be calculated for several values for λ from discrete simulations.

TPF is a more efficient method that approximates the non-linear response of the property  in eq 5 between the neutral (N) and the charged (C) end states from simulations of the end states only through^23^
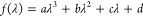
7with the parameters *a*, *b*, *c*, *d* fitted to the first and second derivatives of the free energy with respect to the neutral (λ = 0) and the charged (λ = 1) end states
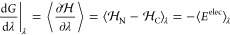
8
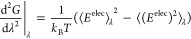
9

The quantity for Δ*G*_N>C_^TPF^ was averaged over both dimers of the protease.

#### Linear Interaction Energy

The simulations that were executed for the TPF approach were analyzed to extract polar and nonpolar averages (Coulomb and VdW interaction energies). Binding free energies were then calculated using the approach of LIE.^41,42^ These were obtained from the averages as
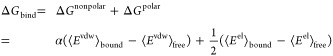
10with α being an empirical coefficient, which was found to be 0.161.^43^ All energies were averaged over both dimers of the protease.

#### Corrections for Free Energies of Charging

The free energies calculated using the TPF procedure are artifacted raw values. The reason is that the electrostatic potentials deviate from the “correct” potentials since they were calculated using a non-Coulombic interaction function (Barker Watts Reaction Field Method^32^) under PBCs. These deviations translate directly into free energies. In the case of charged amino acids, these artifacts do not cancel upon application of a thermodynamic cycle. Typically, these artifacts are of considerable size when free energies of ligand binding are calculated.^44,45^ In these cases, a ligand was simulated in the bound state and in the unbound state, that is, free in solution. Because of the different solvation states, the deviations of the electrostatic potentials between a ligand free in solution and a ligand bound to a solvent-excluded host cavity are not similar and do not cancel upon application of a thermodynamic cycle. In this study, free energies of charging are calculated for point mutations inside the protein, with both legs of the thermodynamic cycle being the protein in the bound and in the unbound (protein without substrate, apo) states. The corrections were thus expected to be smaller in total size but still significant. An exact description of the applied methodology for the post-simulation corrections can be found elsewhere.^45^ In short, the deviation of the “correct” charging free energies can be calculated using continuum electrostatics methods^46^ and analytical models.^47^ These corrections must account for (i) the deviation of the solvent polarization around the charged group of atoms due to the use of a microscopic system in combination with cut-off truncation and a reaction-field correction relative to the “correct” polarization in a macroscopic, non-periodic, and fully Coulombic environment, (ii) the deviation of the solvent-generated electric potential in a microscopic box under PBCs relative to the “correct” potential under full Coulombic, macroscopic, and non-PBCs, (iii) the inaccurate electrostatic interactions between the charged group of atoms and other solute atoms due to the usage of cut-off truncation in combination with a reaction field correction, and (iv) an inaccurate dielectric permittivity of the employed solvent model.

#### *In Vitro* Experiments

All chemicals were purchased from ROTH (Carl Roth GmbH + Co. KG, Karlsruhe, Germany) and were of analytical grade; primers were ordered from Sigma-Aldrich (Merck KGaA; Darmstadt, Germany) and HPLC-purified.

Saturation mutagenesis with degenerate primers, designed to create all possible 19 amino acid substitutions at one site in the protein, was performed with circularly permutated Caspase-2 (cp-Casp2)^48^ with amino acid mutations E105V G171D (sequence can be found in the Supporting Information) in a pACYCDuet-1 vector as a template.

For site-specific random mutations, the NEB Q5 Site-Directed Mutagenesis Kit (New England BioLabs; Ipswich, MA, USA) was used. Mutations at positions V105 and D171 were inserted sequentially in two separate PCRs.

Primers were designed with the degenerate codon NNS at the site of mutation, which generates all 20 amino acids with 32 codons and reduces codon redundancy.

For the mutation at position V105, the forward primer TCGTTGTAAAnnsATGAGCGAGTATTG and the reverse primer TGAAATTCTGTACCCGGTG were used. The ligated product was purified and used as a template for the following mutagenesis to insert mutations at position D171. The forward primer CATTTTACCnnsGAAAAAGAACTG and the reverse primer AACATTGCTCAGAACCAG were used. Sequencing of the pooled gene library showed a clear preference for the nucleotide G at the degenerate position which only produced a reduced sequence space. An additional set of primers was used to exclude codons already found in the previous PCR. The forward primers CATTTTACChhcGAAAAAGAACTG and CATTTTACChhgGAAAAAGAACTG and for both the same reverse primer AACATTGCTCAGAACCAG were used.

Following this, a KLD (kinase, ligase, dpnI) reaction was obtained. NovaBlue heat shock competent cells (Novagen, Merck KGaA; Darmstadt, Germany) were transformed with the ligated product and the cells were diluted into an overnight culture which was used for DNA preparation. Sequencing of the pooled gene library from primers with NNS, HHC, and HHG codons was used to control the quality of the library before selection. All nucleotides were represented in the first two degenerate positions to theoretically produce all 400 possible variants.

*Escherichia coli* BL21(DE3) pyr cells that contained an essential substrate protein that was blocked with a Caspase cleavage tag^48^ with P1′ Thr and Pro were transformed with the gene library. The selection was executed either in a liquid assay or as a plate assay.^49^ The DNA of 161 single colonies was analyzed by sequencing, detecting combinations of mutations in active cp-Casp2 variants.

## Results and Discussion

### Free-Energy Calculations

The Lennard–Jones contributions to the free energies were calculated from two independent simulations using two different reference states, RDR and R2R. Most of the pairwise results from both reference states showed differences in the free energies that are above the thermal energy (*k*_B_*T*). To evaluate the relevance of individual values, the percentage of contributing conformations was calculated for both reference states over all five individual structures that were used for the fitting procedure in the unbound and bound states. A contributing conformation was defined as a snapshot where comparison of the free energies with the energies after OSP gave Δ*G* > Δ*H* (Tables 1 and 2). In general, the, “smaller” reference state RDR (1 soft atom, bond length 0.153 nm) sampled more relevant conformations for amino acids with low molecular weights, while the, “bigger” reference state R2R (2 soft atoms, bond lengths 0.252 and 0.351 nm) was more appropriate for heavier amino acids. This is also reflected in the huge deviations between the free energies calculated from both reference states. For example, the calculated ΔΔ*G* for Arg in the E105 site with Ile in the P1′ site is 110.2 kJ/mol for the state RDR, with the percentage of relevant conformations being 0.5%. However, the equivalent numbers for the R2R state are −1.9 kJ/mol and 16.2%. The least contributing frames from both reference states were counted for the hydrophobic and aromatic amino acids Phe, Tyr, and Trp, making it the most challenging to calculate meaningful free energies for these amino acids. In particular, the free energies obtained for Trp at position 105 with Ile at the P1′ site are seemingly artificially large (Table 1). For future work, a more specific larger reference state may be designed to improve predictions for these amino acids.

**Table 1:** Free Energies from One-step Perturbation for Both Reference States in the E105 Site^a^

P1′	**mut at 105**	**RDR p_contr**	**RDR** ΔΔ*G*_R>N_	**R2R p_contr**	**R2R** ΔΔ*G*_R>N_
**Ile**	Ala	89.7	–16.2	28.52	1.0
	Arg	0.52	110.2	16.15	–1.9
	Asn	26.99	–5.1	11.18	3.4
	Asp	30.38	–4.2	11.77	–4.6
	Cys	0.89	–10.5	2.84	–16.3
	Gly	2.11	–1.8	1.66	–6.9
	Gln	1.08	6.0	5.81	6.6
	Glu	17.99	–6.0	17.97	–1.2
	HisA	1.23	–24.3	6.03	–8.8
	HisB	0.84	–4.2	5.51	–7.0
	Ile	2.75	–14.7	3.00	–1.2
	Leu	4.97	–7.0	1.02	2.2
	Lys	0.25	–5.8	1.45	0.3
	Met	0.59	–20.7	5.18	2.4
	Phe	0.35	44.5	0.98	10.2
	Ser	65.53	3.7	16.27	–12.6
	Thr	26.89	–2.2	16.59	–2.6
	Trp	0.45	–79.2	0.30	67.2
	Tyr	0.56	–9.1	0.37	6.9
	Val	11.52	–1.2	8.14	–1.2
**Pro**	Ala	76.64	–9.9	25.70	–8.5
	Arg	0.60	162.3	18.84	–20.0
	Asn	8.47	–0.3	10.91	–11.8
	Asp	10.44	2.7	8.06	4.0
	Cys	0.78	4.0	6.13	0.6
	Gly	1.74	0.1	0.99	6.5
	Gln	0.45	4.3	6.03	14.6
	Glu	7.49	7.7	19.62	5.6
	HisA	5.28	15.0	17.22	–5.1
	HisB	16.48	–2.0	19.60	–8.4
	Ile	0.78	–7.2	3.13	3.0
	Leu	4.37	–1.4	0.67	16.2
	Lys	0.67	–69.4	8.21	–4.1
	Met	1.00	–16.1	7.58	–15.2
	Phe	0.39	36.0	8.43	–5.1
	Ser	31.17	2.8	10.00	8.0
	Thr	14.64	6.2	3.22	3.3
	Trp	0.47	–187.3	4.55	–21.7
	Tyr	0.45	485.5	1.91	12.0
	Val	6.51	2.3	13.28	–3.1

a Free energies are reported for two P1′ amino acids, Ile and Pro. Next to the ΔΔ*G* values (reported in kJ/mol), the percentage of contributing frames are reported as an average over both bound and unbound states. HisA: N^δ1^-H tautomer; HisB: N^ϵ2^–H tautomer.

**Table 2:** Free Energies from One-step Perturbation for Both Reference States in the G171 Site^a^

P1′	**mut at 171**	**RDR p_contr**	**RDR** ΔΔ*G*_R>N_	**R2R p_contr**	**R2R** ΔΔ*G*_R>N_
**Ile**	Ala	64.84	–10.6	4.78	–6.4
	Arg	0.31	5.2	2.11	–2.0
	Asn	28.96	4.0	11.41	1.5
	Asp	26.68	–12.9	12.18	–5.1
	Cys	1.13	–4.3	1.89	2.3
	Gly	8.45	3.8	4.24	0.7
	Gln	4.51	–13.0	11.45	–7.8
	Glu	5.20	–10.4	13.13	–8.6
	HisA	2.29	5.4	10.22	2.5
	HisB	2.27	3.0	10.28	2.7
	Ile	3.36	1.9	3.76	1.6
	Leu	10.75	4.0	6.06	–1.2
	Lys	1.29	–3.4	8.88	–2.2
	Met	3.46	3.2	11.04	0.8
	Phe	0.42	7.9	3.93	3.7
	Ser	53.00	–0.05	8.85	2.3
	Thr	31.76	1.3	6.20	1.8
	Trp	0.22	0.3	0.68	1.3
	Tyr	0.32	10.2	2.44	17.2
	Val	10.02	–4.9	5.58	1.5
**Pro**	Ala	67.34	13.8	41.12	28.6
	Arg	0.33	–3.3	2.79	4.5
	Asn	38.82	1.0	34.36	19.6
	Asp	44.67	2.4	32.47	6.2
	Cys	48.66	4.7	7.22	23.5
	Gly	2.34	2.4	1.77	2.9
	Gln	26.00	1.0	8.23	12.6
	Glu	25.12	–2.9	12.90	2.9
	HisA	6.08	–1.4	17.49	12.7
	HisB	7.10	1.2	21.91	13.6
	Ile	11.04	–7.6	3.36	11.6
	Leu	25.86	–5.4	8.82	10.3
	Lys	2.55	–6.9	12.12	10.8
	Met	8.28	2.0	19.70	12.4
	Phe	1.18	–17.4	8.95	8.9
	Ser	58.28	7.7	36.09	25.4
	Thr	39.23	1.2	13.80	19.4
	Trp	0.26	–26.5	4.64	6.0
	Tyr	0.46	–14.8	4.86	4.5
	Val	34.84	–4.7	6.79	17.0

a Free energies are reported for two P1′ amino acids, Ile and Pro. Next to the ΔΔ*G* values (reported in kJ/mol), the percentage of contributing frames are reported as an average over both bound and apo states.HisA: N^δ1^–H tautomer; HisB: N^ϵ2^–H tautomer.

The Coulomb contributions to the free energies were calculated from the average energies and the fluctuations (rmsd) of the energies in the charged and the neutral states using the TPF method. For the four charged amino acids Arg, Asp, Glu, and Lys, a sole TPF analysis is not sufficient because of the different net charge of the system in the charged and the neutral states. In these cases, the TPF results are raw values that need to be corrected by a set of correction terms Δ*G*_pol_, Δ*G*_dir_, and Δ*G*_dsm_ for both bound and apo states (see Tables 3 and 4). The total sizes of the corrections for both sites, E105 and G171, differ significantly in size. These differences can be explained structurally: while the site G171 is not directly interacting with the substrate but is highly solvated, the environments between both states, bound and apo, are highly similar and the main artifacts cancel. However, the site E105 is in close proximity to the binding site. In this position, this site is exposed to a higher negative-charge density (P3–P1 sites of the substrate bear negative charges) in case of the bound state compared to the apo state where the binding pocket is more solvated. This can be found in the bigger Δ*G*_dsm_ values for the apo states compared to the bound states for the E105 site.

**Table 3:** Correction Terms for Free Energies of Charging, Δ*G*_pol_ + Δ*G*_dir_, Δ*G*_dsm_ and their Sum for the Four Charged Amino Acids Arg, Asp, Glu, and Lys^a^

		**bound**			**unbound**			**Tot**
P1′	**mut at 105**	Δ*G*_pol_ + Δ*G*_dir_	**Δ*G***_**dsm**_	**sum**	Δ*G*_pol_ + Δ*G*_dir_	**Δ*G***_**dsm**_	**sum**	**Δ*G***_**cor**_
**Ile**	Arg	3.3	–17.3	–14.0	13.0	–24.6	–11.6	–2.4
	Asp	4.1	14.8	18.9	–26.7	17.3	–9.4	28.3
	Glu	11.9	14.3	26.2	–19.4	16.6	–2.8	29.0
	Lys	7.0	–17.1	–10.1	15.6	–18.2	–2.6	–7.5
**Pro**	Arg	–1.2	–18.6	–19.8	9.9	–22.7	–12.8	–7.1
	Asp	4.9	16.5	21.4	–14.2	16.7	2.5	18.9
	Glu	4.5	14.0	18.5	–23.7	16.6	–7.1	25.6
	Lys	1.4	–18.6	–17.2	15.2	–19.7	–4.5	–12.7
**at 171**								
**Ile**	Arg	–7.5	–53.7	–61.2	–5.5	–54.6	–60.1	–1.1
	Asp	6.8	57.5	64.3	6.2	57.0	63.2	1.1
	Glu	5.2	58.0	63.1	6.1	56.8	62.9	0.2
	Lys	–6.7	–52.4	–59.1	–4.9	–51.9	–56.8	–2.3
**Pro**	Arg	–7.7	52.5	60.2	–5.6	–54.2	–59.8	–0.4
	Asp	5.2	56.9	63.9	5.6	57.4	63.1	0.8
	Glu	6.2	56.9	63.1	3.5	58.0	61.5	1.6
	Lys	–3.9	–54.4	–58.2	–1.0	–56.0	–57.0	–1.2

a The corresponding correction terms were calculated for both mutation sites, E105 and G171, as well as both P1′ amino acids, **Ile** and **Pro**. The total correction for the TPF results was deduced from the corrections in the bound and the unbound states of the protease. All values are reported in kJ/mol.

**Table 4:** Results From the TPF Calculations for the Four Charged Amino Acids Arg, Asp, Glu, and Lys^a^

P1′	**mut at 105**	Δ*G*_N>C,raw_	Δ*G*_cor_	Δ*G*_N>C_
**Ile**	Arg	10.2	–2.4	7.9
	Asp	–19.4	28.3	8.9
	Glu	–19.3	29.0	9.7
	Lys	4.4	–7.5	–3.2
**Pro**	Arg	9.3	–7.1	2.2
	Asp	–23.4	18.9	–4.5
	Glu	–42.0	25.6	–16.4
	Lys	29.8	–12.7	17.1
**at 171**				
**Ile**	Arg	–0.3	–1.1	–1.4
	Asp	5.3	1.1	6.4
	Glu	–0.8	0.2	–0.6
	Lys	0.7	–2.3	–1.6
**Pro**	Arg	4.2	–0.41	3.8
	Asp	–4.7	0.85	–3.9
	Glu	–4.6	1.63	–2.9
	Lys	–1.3	–1.21	–2.5

a The raw results from the TPF analysis were corrected for the spurious free energies of charging to yield correct TPF results. All values are reported in kJ/mol.

Table 5 combines the OSP results from the respective reference state with higher percentages of relevant conformations with the TPF results to yield the final free energies of binding. The individual OSP results are shown for the reference states with a higher number of contributing frames. OSP results for Glu (105 site) and Gly (171 site) are shown for both reference states in order to be able to calculate relative free energies for the individual mutations Glu105Xxx and Gly171Xxx through cycle closure (see next section).

**Table 5:** Free Energies from OSP, TPF, and their Sum to Yield Final Free Energies of Binding Relative to the Reference State (ΔΔ*G*^bind^).^a^

**mut at 105**	**P1’ = Ile**			**P1’ = Pro**		
mut	OSP	TPF	ΔΔ*G*^bind^	OSP	TPF	ΔΔ*G*^bind^
Ala	–16.2		–16.2	–9.9		–9.9
Arg	–1.9	7.8	6.0	–20.0	2.2	–17.8
Asn	–5.1	–5.3	–10.4	–11.8	–8.0	–19.8
Asp	–4.2	8.9	4.8	2.7	–4.5	–1.8
Cys	–16.3	11.0	–5.4	0.6	–15.9	–15.3
Gly	–1.8		–1.8	0.1		0.1
Gln	6.6	–3.6	3.0	14.6	–5.8	8.7
Glu (RDR)	–6.0	9.7	3.7	7.7	–16.4	–8.7
Glu (R2R)	–1.2	9.7	8.5	5.6	–16.4	–3.1
HisA	–8.8	2.6	–6.2	–5.1	0.9	–4.2
HisB	–7.0	–1.0	–8.0	–8.4	1.7	–6.7
Ile	–1.2		–1.2	3.0		3.0
Leu	–7.0		–7.0	–1.4		–1.4
Lys	0.3	–3.2	–2.9	–4.1	17.1	13.0
Met	2.4	–0.2	2.3	–15.2	–0.1	–15.4
Phe	10.2	–0.3	9.9	–5.1	7.4	2.2
Ser	–12.6	–1.1	–13.7	2.8	1.4	4.2
Thr	–2.6	–0.9	–3.5	6.2	–3.4	2.9
Trp	–79.2	3.4	–75.8	–21.7	0.4	–21.3
Tyr	–9.1	3.4	–5.7	12.0	–6.1	5.9
Val	–1.2		–1.2	–3.1		–3.1
**at 171**	**P1’ = Ile**			**P1’ = Pro**		
mut	OSP	TPF	ΔΔ*G*^bind^	OSP	TPF	ΔΔ*G*^bind^
Ala	–10.6		–10.6	13.8		13.8
Arg	–2.1	–1.4	–3.4	4.1	3.8	7.9
Asn	4.0	–1.0	3.0	1.0	–0.3	0.6
Asp	–12.9	6.4	–6.5	2.4	–3.9	–1.5
Cys	2.3	3.8	6.1	4.7	–1.7	3.0
Gly (RDR)	3.8		3.8	2.4		2.4
Gly (R2R)	0.7		0.7	2.9		2.9
Gln	–7.8	0.1	–7.7	1.0	–0.8	0.2
Glu	–8.6	–0.6	–9.3	–2.9	–2.9	–5.8
HisA	2.6	–0.9	1.7	–1.4	–0.1	–1.5
HisB	2.7	1.5	4.1	13.6	–0.5	13.0
Ile	1.6		1.6	–7.6		–7.6
Leu	4.0		4.0	–5.4		–5.4
Lys	–2.2	–1.6	–3.8	10.8	–2.5	8.3
Met	0.8	0.7	1.4	12.4	–1.2	11.3
Phe	3.7	–0.1	3.6	8.9	0.8	9.6
Ser	–0.1	0.1	0.7	7.7	1.0	8.7
Thr	1.3	–4.1	–2.8	1.2	1.4	2.5
Trp	1.3	–1.7	–0.5	6.0	0.7	6.7
Tyr	17.2	0.2	17.4	4.5	–1.9	2.7
Val	–4.9		–4.9	–4.7		–4.7

a All results are reported in kJ/mol for both mutation sites and both P1′ amino acids. HisA: N^δ1^–H tautomer; HisB: N^ϵ2^–H tautomer.

### Comparison to Experiments

From the free energies of binding for the individual amino acids, relative free energies of binding for the mutations Glu105Xxx and Gly171Xxx were calculated and compared to the experimental observations (see Table 6). Note that comparison of these data has to be handled very carefully. A mutant will be found in the experiments if the effect of the point mutation renders the protease more active, unchanged, or only marginally less active. On the other hand, a negative change of the numerically derived binding free energies has to be interpreted as more favorable binding. It follows that an exact match of the simulation and experimental data cannot be expected; however, the overall pattern can be compared. Also, the search space covered in the experiment was 20 × 20 = 400 different combinations of amino acid mutations. The individual mutations of both sites were thus not independent of each other. In the computational analysis, each site was mutated while the other position was kept fixed. Finally, in the experiment, Thr and Pro were chosen as P1′ sites instead of Ile and Pro. Ile and Thr are both β-branched and weak binders of the S1′ site (*K*_M_ of Ile/Thr in cp-Casp2: 71 ± 25/75 ± 25 μM).^7^ As a consequence, the data for these two amino acids will be compared within the following analysis. While it is unfortunate that an exact comparison cannot be made, we chose not to adjust our simulations after the validation experiments were performed but to compare how relevant the predictions are in a realistic experimental setting where the exact relevant experiments may not be accessible.

**Table 6:** Comparison of the Final Results from Simulation and Experiment for Both Sites of Point Mutation and Different P1′ Amino Acids^a^

	**mutation at 105**				**mutation at 171**			
	**P1’ = Ile**		**P1’ = Pro**		**P1’ = Ile**		**P1’ = Pro**	
	sim	exp	sim	exp	Sim	exp	sim	exp
Ala	–19.9	√	–1.2	√	–14.3	√	11.5	√
Arg	–2.5	√	–14.7		–4.1	√	4.9	√
Asn	–14.1	√	–16.7		–0.8		–1.8	√
Asp	1.1		6.9		–10.3		–3.9	√
Cys	–13.8	√	–12.2		5.4	√	0.7	√
Gly	–1.8	√	0.1		0		0	
Gln	–5.5	√	11.8		–8.4		–2.1	√
Glu	0	√	0		–9.9	√	–8.2	√
HisA	–14.7	√	–1.1		0.9		–4.5	
HisB	–16.5	√	–3.6		3.4		10.9	
Ile	–9.7	√	6.1		0.9		–10.0	
Leu	–10.7	√	7.2		0.3		–7.8	
Lys	–11.4		16.1		–4.5	√	5.3	
Met	–6.2	√	–12.3	√	0.8		8.3	
Phe	1.5		5.3		3.0		6.7	
Ser	–22.2	√	12.9		–3.7	√	6.3	
Thr	–11.9	√	11.5		–6.6		0.2	
Trp	–79.5	√	–18.2		–1.2	√	3.8	
Tyr	–9.4	√	9.0		16.8		–0.3	
Val	–9.7	√	0.0	√	–8.7	√	–7.1	√

a Relative substrate binding free energies (“sim” column) are reported for the mutations Glu105Xxx and Gly171Xxx (relative to the unmated protein). In the columns with the experimental results, check marks/horizontal bars mark mutations that were found/not found in the screening. All values are reported in kJ/mol. HisA: N^δ1^–H tautomer; HisB: N^ϵ2^–H tautomer.

All effects of the mutations in both sites were compared with Ile and Pro in the P1′ site. With Ile in the P1′ site, only three mutations for the 105 site were not found in the experiment–Asp, Lys, and Phe. Two of these mutations were calculated to be unfavorable, while Lys was calculated to be favorable for binding of the substrate (−11.4 kJ/mol). All amino acids that were found in the experimental random mutagenesis were also calculated to have favorable binding free energies. The situation is more complex with P1′ Pro. Here, only three mutations were found in the experiment: Ala, Met, and Val. All three amino acids were also attributed with favorable binding free energies. However, five amino acids were calculated to have favorable changes in free energies of binding, but were not found in the experiment. For mutations of the 171 site, nine amino acids were experimentally found with Ile in the P1′ site. Seven of these were also favorable in the free-energy calculations. Six of ten amino acids that were not found in the experiment showed unfavorable binding free energies. With P1′ Pro, again only three amino acids were found in the experiment, Asp, Glu, and Val. All three were also associated with favorable free energies. 10 of 15 amino acids that were not found also had unfavorable free energies attributed to them. To validate the predictive power of the computationally derived free energies, Table 7 compares these data in three cross tables: two for the data with P1′ = Ile and P1′ = Pro, respectively; another cross table shows both data sets combined. From these numbers, the Matthews correlation coefficient (MCC) was calculated to validate the predictive power of the computational data. Both experiment and simulation come to a comparable conclusion for a majority of the amino acids: with Ile in the P1′ site, the predictive power of computational saturation mutagenesis is very good. 61% of the amino acids showed favorable free energies and were also found in the experiment. 22% were calculated to have unfavorable free energies attributed to them and were not found in the experiment. Only 17% of the amino acids were false positives or false negatives. The MCC was found to be 0.63. With Pro in the P1′ site, the predictive power was found to be less: 22% of the amino acids had favorable free energies attributed to them but were not found in the experiment. However, 47% of the amino acids were predicted to have unfavorable free energies and were also not found in the experiment. The MCC for the Pro data was found to be 0.38. A possible explanation for the higher rate of false positives in the Pro data is the following: experimental findings reflect the effects on binding and cleavage, while the computational data focus on free energies connected to substrate binding only. With Pro being a highly unfavorable substrate for the S1′ pocket, the ligand may be able to bind but the probability of reaching an orientation in a catalytically active pose remains low. In the combined data set, 42% of the amino acids showed favorable free energies and were found experimentally; 40% showed unfavorable free energies and were not found experimentally. The remaining set of amino acids had favorable free energies attributed but were not found–or *vice versa*. For the combined data, the MCC was found to be 0.55. The data show that 76% of the predictions by this method are correct. For the incorrect predictions, it is more likely to predict false positives than false negatives. This reflects the fact that the calculation of binding affinities does give an insight into the actual binding process but gives no information if cleavage is likely to happen.

**Table 7:** Experimental Findings (Mutation Found/Not Found) and Results from Simulations (Mutation Favorable/Unfavorable) Represented in a Cross Table^a^

	found (%)	not found (%)
**Ile**		
fav	61.1	13.8
unfav	2.8	22.2
**Pro**		
fav	22.2	22.2
unfav	8.3	47.3
**All**		
fav	41.7	18.1
Unfav	5.6	34.7

a The numbers were calculated for all results from both mutation sites and P1′ amino acids.

### Comparison to the Linear Interaction Energy Approach

The predictions based on the OSP + TPF approach were evaluated against another established end-point method. Hence, the trajectories simulated for the TPF approach were reused to extract polar and nonpolar energies to calculate binding free energies *via* the LIE approach. Mind that trajectories for TPF were only generated for polar amino acids; hence, this comparison is restricted to these. Table 8 reports binding free energies from LIE and OSP + TPF. Energies for the individual bound and unbound states as well as free energies from LIE are reported in Tables S1–S4 in the Supporting Information. The agreement between the two approaches is moderate with a mean-absolute difference (MAD) of 5–22 kJ/mol and Pearson correlation coefficient (*r*) between −0.31 and 0.41 for the four combinations of the mutation site and P1′ residue. For each combination of mutation sites and the P1′ site, MCCs were calculated to quantify the agreement with the experimental data for each method. These MCC values are summarized in Table 9. The combined OSP + TPF approach outperformed the LIE method in every case. While the LIE approach requires approximately half the simulation time compared to the OSP/TPF approach, an MCC value close to zero was obtained for three of the four combinations of mutation sites and the P1′ residues. Only for the mutation site G171 and the Pro residue at P1′ is an MCC value of 0.29 obtained, which is still considerably worse than the value obtained with the OSP/TPF approach, at 0.56.

**Table 8:** Relative Binding Free Energies From the LIE Method and the Combined OSP + TPF Results in Comparison Against Experimental Findings^a^

**mut at 105**	**P1’ = Ile**			**P1’ = Pro**		
mut	LIE	OSP + TPF	exp	LIE	OSP + TPF	exp
Arg	1.4	–2.5	√	–18.5	–14.7	
Asn	–12.3	–14.1	√	–20.4	–16.7	
Asp	34.0	1.1		–47.1	6.9	
Cys	5.1	–13.8	√	–11.8	–12.2	
Gln	–8.9	–5.5	√	–30.9	11.8	
Glu	0.0	0.0	√	0.0	0.0	
His	–1.2	–15.6	√	–23.2	–2.4	
Lys	–20.6	–11.4		–11.0	16.1	
Met	–0.9	–6.2	√	–24.8	–12.3	√
Phe	–6.9	1.5		–29.7	5.3	
Ser	–10.5	–22.2	√	–22.9	12.9	
Thr	–10.2	–11.9	√	–19.0	11.5	
Trp	–15.8	–79.5	√	–30.5	–18.2	
Tyr	2.8	–9.4	√	–20.3	9.0	
MAD		13.3			22.0	
R		0.41			–0.06	
**at 171**	**P1′ = Ile**			**P1′ = Pro**		
mut	LIE	OSP + TPF	exp	LIE	OSP + TPF	exp
Arg	4.8	–4.1	√	–2.2	4.9	√
Asn	–0.1	–0.8		–4.4	–1.8	√
Asp	4.2	–10.3		–0.5	–3.9	√
Cys	8.8	5.4	√	7.1	0.7	√
Gln	–0.2	–8.4		2.4	–2.1	√
Glu	–0.3	–9.9	√	–0.1	–8.2	√
His	0.4	2.2		3.8	3.2	
Lys	–4.1	–4.5	√	–2.8	5.3	
Met	1.6	0.8		–14.2	8.3	
Phe	–0.1	3.0		4.2	6.7	
Ser	3.4	–3.7	√	–2.1	6.3	
Thr	–6.7	–6.6		4.6	0.2	
Trp	–1.8	–1.2	√	0.8	3.8	
Tyr	–0.2	16.8		1.6	–0.3	
MAD		5.5			6.0	
R		0.15			–0.31	

a All free energies are reported in kJ/mol for both mutation sites and both P1′ amino acids. MAD: mean average deviation between LIE and OSP + TPF approach; r: Pearson correlation coefficient between LIE and OSP + TPF approach.

**Table 9:** MCC to Compare the Combined OSP + TPF Method Against the LIE Approach for the 14 Compounds in Table 8^a^

site	P1′	LIE	OSP + TPF
E105	**Ile**	0.03	0.78
E105	**Pro**	0.00	0.31
G171	**Ile**	0.00	0.34
G171	**Pro**	0.29	0.56

a For each combination of mutation site and P1′, OSP + TPF outperformed LIE.

## Conclusions

In the presented work, an efficient method for *in silico* saturation mutagenesis was qualitatively validated against experimental data. Two sites in the binding pocket were screened in human Caspase-2. The entire procedure was repeated using two substrates with different amino acids at the P1′ position. Nonpolar contributions to the free energies of binding were calculated from two different reference states, each in the bound and the unbound protease and the electrostatic contribution was added using the TPF method. After judicious application of a thermodynamic cycle, relative binding free energies compared to the natural form of the protease were obtained. These free energies were compared to data from experimental saturation mutagenesis and selection with an auxotrophic *E.coli* strain. The overall agreement was very good, with the MCC being 0.55. It is obvious that it is much more likely that the *in silico* saturation mutagenesis predicts false positives than false negatives. This reflects that a ligand that is a good binder does not necessarily undergo cleavage. Although the intrinsic reactivity of the mutated protease is believed to be the same toward substrates with different P1′ sites, our method is not fit to discriminate bound states that undergo cleavage from bound states where cleavage is unlikely. However, this is acceptable since the general aim of this method is to narrow the sequence space of a protein of interest a priori. It can be concluded that free energies that were derived through the efficient OSP + TPF approach can be used as a powerful predictor to qualify the effect of single point mutations on protease–substrate cleavage. Thus, we believe that this approach represents an efficient and powerful tool for rational-design strategies.

## Supporting Information Available

Amino acid sequence of cpCasp2 and free energy estimates obtained from the LIE approach with mutations at sites E105 and G171 and with Ile and Pro at the substrate site P1’ (PDF)
