# Supplementary material for: Efficient In Silico Saturation Mutagenesis of a Member of the Caspase Protease Family
Source: J Chem Inf Model. 2021 Feb 11;61(3):1193–203. doi: 10.1021/acs.jcim.0c01216 (PMC8023567; doi:10.1021/acs.jcim.0c01216)
Supplement: Supplementary file 1 — ci0c01216_si_001.pdf [file ci0c01216_si_001.pdf]

Supporting Information: Efficient *In Silico* Saturation Mutagenesis of a Member of the *Caspase* Protease Family

Christoph Öhlknecht,[†,‡] Sonja Katz,[†,‡] Christina Kroess,[¶,‡] Bernhard Sprenger,[¶,‡] Petra Engele,[¶,‡] Rainer Schneider,[¶] and Chris Oostenbrink[*,†]

†*Institute of Molecular Modeling and Simulation, University of Natural Resources and Life Sciences, Vienna, Austria*

‡*Austrian Centre of Industrial Biotechnology, Petersgasse 14, Graz, Austria*

¶*Institute of Biochemistry and Center of Molecular Biosciences Innsbruck, University of Innsbruck, Austria*

E-mail: chris.oostenbrink@boku.ac.at

Figure S1: Amino-Acid Sequence of cp-Casp2 with N-terminal His Tag, GS linker, CEESA and E105V and G171D substitution (SEQ ID No. 253)

CEESA (above residues CEESA); *GS linker* (below residues SP)

cp-Casp2	MHHHHHHGKNHAGSPGCEESAAGKEKLPKMRLPTRSDMICGYACLKGTAA	50
cp-Casp2	MRNTKRGSWYIEALAQVFSERACDMHVADMLVKVNALIKDREGYAPGTEF	100
cp-Casp2	HRCKVMSEYCSTLCRHLYLFPGHPPTGSGPVCLQVKPCTPEFYQTHFQLA	150
cp-Casp2	YRLQSRPRGLALVLSNVHFTDEKELEFRSGGDVDHSTLVTLFKLLGYDVH	200
cp-Casp2	VLCDQTAQEMQEKLQNFAQLPAHRVTDSCIVALLSHGVEGAIYGVDGKLL	250
cp-Casp2	QLQEVFQLFDNANCPSLQNKPKMFFIQACRGDETDRGVDQQD	292

E105V ↓ (at V, residue 105); *G171D* ↓ (at D, residue 171)

Table S1: Extracted polar and nonpolar energies from the TPF trajectories and free energies from the LIE method for the E105 mutation and P1'=Ile. All values are reported in kJ/mol.

Mut	$\langle E^{el}_{apo} \rangle$	$\langle E^{vdw}_{apo} \rangle$	$\langle E^{el}_{lig} \rangle$	$\langle E^{vdw}_{lig} \rangle$	LIE
Arg	-871.37	-89.37	-852.42	-92.21	9.03
Asn	-665.68	-51.41	-678.73	-39.35	-4.60
Asp	-1223.10	-22.74	-1140.36	-21.01	41.65
Cys	-290.97	-53.63	-264.61	-56.20	12.77
Gln	-657.75	-73.34	-660.36	-72.65	-1.19
Glu	-1311.72	-33.29	-1299.73	-22.89	7.66
HisA	-200.21	-93.09	-171.90	-100.59	12.95
HisB	-260.05	-93.73	-262.27	-87.25	-0.08
Lys	-982.84	-66.61	-1010.21	-61.77	-12.91
Met	-73.71	-115.36	-57.83	-122.71	6.77
Phe	-19.24	-148.96	-18.60	-146.08	0.78
Ser	-177.16	-24.28	-182.49	-25.57	-2.87
Thr	-154.55	-45.36	-159.72	-44.78	-2.49
Trp	-143.86	-184.88	-159.32	-187.44	-8.14
Tyr	-301.66	-146.49	-283.48	-138.17	10.42

Table S2: Extracted polar and nonpolar energies from the TPF trajectories and free energies from the LIE method for the E105 mutation and P1'=Pro. All values are reported in kJ/mol.

Mut	$\langle E^{el}_{apo} \rangle$	$\langle E^{vdw}_{apo} \rangle$	$\langle E^{el}_{lig} \rangle$	$\langle E^{vdw}_{lig} \rangle$	LIE
Arg	-869.56	-92.42	-854.19	-81.03	9.51
Asn	-643.42	-51.82	-626.20	-58.32	7.57
Asp	-1252.19	-5.54	-1292.90	2.41	-19.08
Cys	-286.77	-58.28	-254.62	-57.60	16.18
Gln	-654.18	-72.90	-660.20	-72.15	-2.89
Glu	-1234.53	-36.43	-1180.39	-30.63	28.00
HisA	-204.49	-89.75	-198.68	-87.32	3.29
HisB	-272.79	-90.54	-260.75	-88.34	6.37
Lys	-1045.93	-60.50	-1011.15	-62.71	17.03
Met	-62.55	-128.33	-57.81	-123.23	3.19
Phe	-17.55	-154.52	-23.31	-147.39	-1.74
Ser	-188.67	-20.34	-178.57	-20.16	5.08
Thr	-165.12	-40.58	-147.50	-39.56	8.97
Trp	-158.20	-165.02	-162.20	-167.95	-2.4
Tyr	-289.64	-139.99	-274.31	-139.76	7.70

Table S3: Extracted polar and nonpolar energies from the TPF trajectories and free energies from the LIE method for the G171 mutation and P1'=Ile. All values are reported in kJ/mol.

Mut	$\langle E_{apo}^{el} \rangle$	$\langle E_{apo}^{vdw} \rangle$	$\langle E_{lig}^{el} \rangle$	$\langle E_{lig}^{vdw} \rangle$	LIE
Arg	-917.79	-57.34	-910.22	-51.34	4.75
Asn	-664.39	-36.07	-665.79	-32.06	-0.06
Asp	-1513.41	36.22	-1505.20	37.01	4.23
Cys	-315.58	-43.34	-295.22	-52.00	8.80
Gln	-667.49	-56.25	-668.42	-54.62	-0.20
Glu	-1504.26	17.01	-1505.07	17.79	-0.28
HisA	-213.20	-68.99	-215.14	-65.22	-0.37
HisB	-287.39	-69.56	-285.82	-70.56	0.62
Lys	-1048.83	-37.04	-1056.49	-38.43	-4.05
Met	-64.58	-102.48	-61.55	-101.80	1.62
Phe	-29.53	-120.15	-29.49	-121.06	-0.13
Ser	-186.98	-15.60	-179.66	-17.13	3.42
Thr	-162.57	-37.87	-180.11	-25.18	-6.74
Trp	-184.38	-149.14	-190.09	-142.74	-1.83
Tyr	-313.70	-99.41	-313.60	-100.82	-0.18

Table S4: Extracted polar and nonpolar energies from the TPF trajectories and free energies from the LIE method for the G171 mutation and P1'=Pro. All values are reported in kJ/mol.

Mut	$\langle E_{apo}^{el} \rangle$	$\langle E_{apo}^{vdw} \rangle$	$\langle E_{lig}^{el} \rangle$	$\langle E_{lig}^{vdw} \rangle$	LIE
Arg	-791.10	-62.05	-794.02	-66.65	-2.20
Asn	-652.55	-50.99	-662.16	-48.69	-4.44
Asp	-1078.95	-16.79	-1082.22	-9.76	-0.51
Cys	-489.67	-60.48	-476.38	-57.66	7.09
Gln	-665.31	-66.00	-662.68	-59.35	2.38
Glu	-1077.79	-23.84	-1078.37	-22.89	-0.13
HisA	-441.59	-66.98	-436.01	-66.38	2.89
HisB	-479.41	-69.55	-470.92	-68.55	4.40
Lys	-854.37	-51.24	-860.06	-51.14	-2.83
Met	-334.64	-86.66	-364.31	-82.43	-14.16
Phe	-354.67	-97.06	-347.34	-94.02	4.15
Ser	-420.03	-40.86	-425.31	-37.21	-2.05
Thr	-428.14	-47.64	-418.93	-47.52	4.62
Trp	-418.79	-115.69	-419.79	-107.73	0.77
Tyr	-485.45	-82.60	-482.26	-82.88	1.55
